# High-risk screening and detection of multidrug-resistant tuberculosis in two prefectures of China: a drug susceptibility surveillance-based secondary data analysis

**DOI:** 10.1080/16549716.2018.1500763

**Published:** 2018-09-11

**Authors:** Zhiqi Yang, Changming Zhou, Zhu Ning, Wei Lu, Qi Zhao, Yi Hu, Vinod K. Diwan, Biao Xu

**Affiliations:** a Department of Epidemiology, School of Public Health and Key Laboratory of Public Health Safety (Ministry of Education), Fudan University, Shanghai, PR China; b Department of cancer prevention, Fudan University Shanghai Cancer Center, Shanghai, PR China; c Zigong Center for Disease Control and Prevention, Zigong, Sichuan, PR China; d Jiangsu Provincial Center for Disease Control and Prevention, Nanjing, Jiangsu, PR China; e Department of Public Health Sciences (Global Health/IHCAR), Karolinska Institutet, Stockholm, Sweden

**Keywords:** Tuberculosis, multidrug-resistance, screening strategy, high-risk groups, population attributable risk

## Abstract

**Background**: In China, less than one-fifth of multidrug-resistant tuberculosis (MDR-TB) cases are detected. MDR-TB screening is conducted amongst the following five high-risk groups of TB patients: chronic cases, close contacts of MDR-TB patients, patients with treatment failure, relapsed and returned patients, and smear-positive patients at the end of the third month of initial treatment.

**Objective**: To estimate the possibility of detecting MDR-TB cases if only the high-risk screening strategy is applied in China.

**Methods**: A secondary analysis was applied to the surveillance-based longitudinal data of all sputum smear-positive TB patients in Prefecture E and Prefecture W of China from 2013 to 2015. The population attributable risk (PAR) was estimated using odds ratios for five risk factors/predictors and exposure proportions amongst all MDR-TB cases.

**Results**: A total of 3513 TB patients (2807 from Prefecture E and 706 from Prefecture W) were included. Males accounted for 77.91% (2737/3513) of the patients. The average age was 52.5 ± 20.0 years old. Overall, 40.34% (71/176) of MDR-TB patients were from the five high-risk groups during the three-year study period. The detected proportion of MDR-TB cases using the high-risk screening strategy was significantly higher in Prefecture E than in Prefecture W. The PAR% for all five risk factors/predictors was 43.4% (95% CI: 24.6–61.7%), 49.9% (95% CI: 31.3–67.0%), and 30.3% (95% CI: 12.9–50.1%) in Prefecture E and 36.6% (95% CI: 10.4–64.5%), 13.3% (95% CI: −1.7–39.7%), and −82.5% (95% CI: −117.5–−11.2%) in Prefecture W in 2013, 2014, and 2015, respectively. The PAR% for the five specific risk factors/predictors ranged from 0.4% (95% CI: −0.2–4.8%) to 21.0% (95% CI: 13.1–30.0%) in these two prefectures.

**Conclusion**: In general, a high-risk screening strategy would miss more than half of the MDR-TB patients because they do not belong to the five high-risk groups.

## Background

Multidrug-resistant tuberculosis (MDR-TB) refers to tuberculosis that is resistant to, at minimum, both rifampicin and isoniazid, which are the two most effective first-line anti-tuberculosis drugs. There were an estimated 490,000 new cases of MDR-TB and an additional 110,000 rifampicin-resistant tuberculosis (RR-TB) cases that were also eligible for MDR-TB treatment globally in 2016 [1]. However, only 153,119 cases of multidrug-resistant TB or rifampicin-resistant tuberculosis (MDR/RR-TB) were notified, and treatment was initiated in 129,689 cases [1]. China has the second highest number of MDR/RR-TB cases globally, with an estimated 58,000 MDR/RR-TB cases in 2016, but only 10,898 cases were laboratory-confirmed [1]. Compared with that in China, the detection rate of MDR-TB is much higher in other countries with high MDR-TB burdens, such as 44% in India, 58% in the Russian Federation, and 233% in South Africa [1]. Considering the large population of MDR-TB patients in China, such a low detection rate is a challenge for the achievement of the ‘End TB’ goals worldwide.

The major barrier to MDR-TB diagnosis is the poor accessibility to universal drug susceptibility testing (DST) in resource-limited settings [2,3]. In China, the basic unit for TB control programmes is set at the county level. The county designates TB dispensaries/clinics to provide TB medical care to all TB patients diagnosed in the county. Constrained by resources and capacity, TB diagnosis at the county level is microscopy-based; thus, DST is not routinely available. TB patients have varying risks of carrying drug-resistant strains. Previous treatment history, close contact with MDR-TB cases, relapses and returns after default, and being smear-positive at the end of the second or third month of an initial treatment regimen are well-established risk factors or risk predictors for MDR-TB [2,4,5]. DST is the gold standard for MDR-TB diagnosis, but only some high-income countries can afford to test all new TB patients for drug susceptibility [4]. Instead, screening for MDR-TB patients amongst high-risk TB patients has been considered a cost-effective approach and has been applied in countries with high TB disease burden [4,6,7].

China started a collaboration with the Global Fund to Fight AIDS, Tuberculosis, and Malaria (Global Fund) on TB control in 2003, which has supported a project on strengthening MDR-TB management since 2006 [8]. In 2009, the Chinese Ministry of Health (now called the National Health Commission of the People’s Republic of China) organized a ministerial meeting in Beijing and agreed to draw up a plan for MDR-TB prevention and control in China, with the aim of providing MDR-TB screening, diagnosis, treatment, and management [9,10]. The diagnosis and treatment of MDR-TB under the national MDR-TB prevention and control programme have been established at the designated TB hospitals in the superior prefectural cities, and the basic units of the TB control programme at the county level are responsible for referring patients suspected of having MDR-TB. Considering the cost-effectiveness, insufficient laboratory facilities, and bio-safety of county TB dispensaries, MDR-TB screening was conducted only amongst five high-risk groups of TB patients under China’s MDR-TB control programme, including (1) chronic TB patients (patients who experienced multiple treatment courses but remained sputum positive) and TB patients for whom retreatment failed; (2) close contacts of MDR-TB cases who developed sputum smear-positive (SS+) TB; (3) TB patients for whom initial treatment failed; (4) relapsed and retuened TB patients; and (5) patients who remained smear-positive at the end of the third month of the initial treatment regimen [7,11,12].

Under the national MDR-TB prevention and control programme, county TB dispensaries are responsible for drug resistance risk assessment, sputum smear testing and bacterial culture [12]. Sputum samples from suspected MDR-TB cases, i.e. the five high-risk groups, are transferred to laboratories of high-level, prefectural designated MDR-TB hospitals for strain identification and DST [12]. Although MDR-TB screening amongst high-risk patients is reasonable and necessary in regions with a high prevalence of drug-resistant TB, this selective screening strategy might reduce the detection rate of MDR-TB cases. A recent study conducted in Shanghai demonstrated that most MDR-TB cases were detected in previously untreated patients because of the large proportion of new TB patients amongst all notified patients, although the MDR-TB prevalence is much lower in new TB patients than in previously treated patients [13]. Therefore, a large number of MDR-TB patients would be missed under the high-risk screening strategy in China [3,5].

There has not been an evaluation of the effect of MDR-TB detection compared with that of the universal DST approach on the screening of five high-risk groups in China. In addition to prioritizing DST and other available resources, identifying the proportion of MDR-TB patients detected through high-risk screening and assessing the contribution of this high-risk screening strategy to MDR-TB detection are important. Unlike high-risk screening, this study was a surveillance-based longitudinal data analysis conducted in two prefectural cities in eastern and western China where all bacteriologically diagnosed TB patients underwent testing for drug susceptibility. The aim of this study was to estimate the possibility of detecting MDR-TB cases if only the above-mentioned five high-risk groups were screened and to evaluate the contribution of each risk factor/predictor to MDR-TB detection by means of the population attributable risk (PAR).

## Methods

### Study sites and study period

According to the geographic distributions, economic conditions, TB control status, and the availability of drug resistance surveillance for all TB patients, two prefectures (Prefecture E and Prefecture W) were predetermined as the study sites. Prefecture E is located in Jiangsu Province in eastern China and has a population of 8.7 million [14], whereas Prefecture W is located in Sichuan Province in western China and has a population of 3.2 million [15]. As one of the pilot sites of the Global Fund, Prefecture E launched the Global Fund to support the MDR-TB control programme from 2009 to 2014; this programme was then launched in Prefecture W from 2012 to 2014. One of the main activities of this project is providing DST for all sputum smear-positive TB patients [10]. After the withdrawal of the Global Fund project in June 2014, the national MDR-TB control programme of China has taken over and continued until now. The routine work of the MDR-TB programme in these two study sites involves sputum culture, strain identification, and DST for all sputum smear-positive patients, including newly diagnosed TB patients and other patients who do not belong to the five high-risk groups. This study incorporated the MDR-TB surveillance data from 2013 to 2015.

### Study participants and data collection

A surveillance-based secondary analysis was conducted using laboratory data of all smear-positive TB patients, including laboratory results of sputum smear microscopy, sputum culture, strain identification, and DST. All notified smear-positive TB patients diagnosed during 2013–2015 in these two study sites were included as study participants. The demographic and diagnostic information of the participants was obtained from the electronic information system of the national TB report and registration, including sex, age, and patient category. This information was routinely recorded by trained health staff in the county TB control programmes. Bacteriological data were obtained from the prefectural TB labs.

### Bacteriological diagnoses

All the laboratory tests were conducted under the national guidelines [7]. Specifically, each participant submitted three sputum samples to be assessed by smear microscopy in the county TB dispensaries. Samples from smear-positive patients were cultured. Culture-positive isolates were delivered to TB labs in high-level prefectural designated hospitals for strain identification and DST. A modified Loewenstein–Jenson medium was used for culture. The agar-proportion method was used in the DST on isoniazid, rifampicin, ofloxacin and kanamycin, and the critical growth proportion for resistance was 1% for each drug.

### Statistical analysis

Pearson’s chi-square test and Fisher’s exact test were used to examine differences in proportions. A binary logistic regression was performed to adjust for confounding factors such as age and sex. Adjusted odds ratios (aORs) and 95% confidence intervals (CIs) were applied to assess the association between the five high-risk factors/predictors and the diagnosis of MDR-TB. The population attributable risk percentage (PAR%) was estimated in this surveillance data analysis, which is applicable to longitudinal data on rare diseases such as MDR-TB. The PAR% was calculated based on crude odds ratios (cORs) for the five risk statuses and exposure proportions amongst all MDR-TB cases. The PAR% reflected the proportion of MDR-TB cases explained by these five risk factors/predictors amongst the overall number of MDR-TB cases [16,17], which could be further used to evaluate the contribution of the high-risk screening strategy to MDR-TB detection. The PAR% was calculated using Levin’s formula [16]:

$$\frac{p\left(r-1\right)}{p\left(r-1\right)+1}\times 100\%$$

in which p is the proportion of TB patients in each risk group, and r is the odds ratio of being diagnosed with MDR-TB. All statistical analyses were performed using SPSS software 22.0 (IBM, Chicago, IL). A two-sided *p* value of <0.05 was considered statistically significant.

### Ethical considerations

All data used in the analysis were anonymous without any identifying information, and informed consent was exempted based on guidelines of the *Ethical Review of Biomedical Research Involving Human Beings* in China [18]. Ethical clearance for the secondary analysis of surveillance data was issued by the Medical Institutional Review Board of the School of Public Health of Fudan University in Shanghai, China.

## Results

### Demographic characteristics of the participants

A total of 3513 sputum smear-positive TB patients were diagnosed in the 3-year period of 2013–2015 in Prefecture E (*n* = 2807) and Prefecture W (*n* = 706), with 2737 (77.91%) males and 776 (22.09%) females. The average age was 52.5 ± 20.0 years old. Less than 20% of the smear-positive TB patients belonged to the five high-risk groups, except for those identified in 2015 in Prefecture W. In Prefecture E, there were 1092, 961, and 754 registered smear-positive patients in 2013, 2014, and 2015, respectively. Meanwhile, in Prefecture W, there were 266, 276, and 164 registered smear-positive patients in 2013, 2014, and 2015, respectively. The absolute number of smear-positive patients decreased significantly from 2014 to 2015 in these two study sites (*p* = 0.014) after completion of the Global Fund-supported project (Table 1).

**Table 1.** Demographic characteristics of sputum smear-positive TB patients in prefecture E and prefecture W from 2013 to 2015.

Characteristics	2013		2014		2015	
	Prefecture E	Prefecture W	Prefecture E	Prefecture W	Prefecture E	Prefecture W
	*n* (%)	*n* (%)	*n* (%)	*n* (%)	*n* (%)	*n* (%)
**Sex**						
Male	824 (75.46)	223 (83.83)	744 (77.42)	227 (82.25)	588 (77.98)	131 (79.88)
Female	268 (24.54)	43 (16.17)	217 (22.58)	49 (17.75)	166 (22.02)	33 (20.12)
**Age (years)**						
<15	2 (0.18)	1 (0.38)	3 (0.31)	0 (0.00)	3 (0.40)	0 (0.00)
15~	125 (11.45)	12 (4.51)	127 (13.22)	15 (5.43)	125 (16.58)	7 (4.27)
25~	132 (12.09)	23 (8.65)	141 (14.67)	31 (11.23)	136 (18.04)	14 (8.54)
35~	81 (7.42)	28 (10.53)	82 (8.53)	29 (10.51)	69 (9.15)	18 (10.98)
45~	142 (13.00)	50 (18.80)	141 (14.67)	74 (26.81)	75 (9.95)	35 (21.34)
55~	200 (18.32)	75 (28.20)	150 (15.61)	51 (18.48)	106 (14.06)	39 (23.78)
65~	218 (19.96)	48 (18.05)	183 (19.04)	53 (19.20)	114 (15.12)	39 (23.78)
75~	192 (17.58)	29 (10.90)	134 (13.94)	23 (8.33)	126 (16.71)	12 (7.32)
**Patient categories ^a^**						
Low-risk patients	916 (83.88)	224 (84.21)	810 (84.29)	245 (88.77)	625 (82.89)	91 (55.49)
Chronic TB patients	1 (0.09)	1 (0.38)	5 (0.52)	0 (0.00)	6 (0.80)	0 (0.00)
Close contacts of MDR-TB cases	14 (1.28)	1 (0.38)	8 (0.83)	0 (0.00)	11 (1.46)	1 (0.61)
Initial treatment failure	18 (1.65)	1 (0.38)	15 (1.56)	1 (0.36)	14 (1.86)	2 (1.22)
Relapsed and returned	143 (13.10)	39 (14.66)	123 (12.80)	30 (10.87)	87 (11.54)	70 (42.68)
Smear-positive at the end of the third month	0 (0.00)	0 (0.00)	0 (0.00)	0 (0.00)	11 (1.46)	0 (0.00)
**Total**	1092 (100.00)	266 (100.00)	961 (100.00)	276 (100.00)	754 (100.00)	164 (100.00)

^a^Low-risk patients: patients who were not from the subsequent five high-risk groups. Chronic TB patients: patients who experienced multiple treatment courses but remained sputum positive, or patients for whom retreatment failed. Close contacts of MDR-TB cases: close contacts of MDR-TB cases who developed sputum smear-positive TB. Initial treatment failed: new patients who were sputum smear-positive at the end of fifth month of treatment. Relapsed and returned: patients had a definite TB history but were cured after standard treatment and were sputum smear-positive again in the current episode, or patients who had ≥1 month’s anti-TB treatment with ≥2 months’ interruption and returned to treatment again. Smear-positive at the end of the third month: patients who remain smear-positive at the end of the third month of an initial treatment regimen.

### Results of sputum culture and DST

Overall, 89.75% (3153/3513) of the total smear-positive TB patients were culture-positive, and 7.83% (275/3513) were culture negative. Another 1.42% (50/3513) did not have a result owing to rejection or failure of culture. The proportions of patients with positive cultures in Prefecture E were 94.05%, 89.91% and 89.12% in 2013, 2014, and 2015, respectively, which were higher than those in Prefecture W (2013: 93.23%; 2014: 85.87%; 2015: 64.02%); however, a statistically significant difference between the two sites was observed only in 2015 (2013: *p* = 0.619; 2014: *p* = 0.059; 2015: *p* < 0.001) (Table 2).

**Table 2.** Laboratory results of sputum culture, strain identification, and drug-susceptibility tests for participants.

Laboratory results	Prefecture E			Prefecture W		
	2013	2014	2015	2013	2014	2015
	*n* (%)	*n* (%)	*n* (%)	*n* (%)	*n* (%)	*n* (%)
**Smear-positive**	1092	961	754	266	276	164
Culture+	1027 (94.05)	864 (89.91)	672 (89.12)	248 (93.23)	237 (85.87)	105 (64.02)
Culture−	54 (4.95)	86 (8.95)	71 (9.42)	16 (6.02)	38 (13.77)	10 (6.10)
Contaminated	11 (1.01)	11 (1.14)	10 (1.33)	2 (0.75)	1 (0.36)	0 (0.00)
Unreported	0 (0.00)	0 (0.00)	1 (0.13)	0 (0.00)	0 (0.00)	49 (29.88)
**Culture-positive**	1207	864	672	248	237	105
M.tb ^a^	975 (94.94)	789 (91.32)	642 (95.54)	241 (97.18)	233 (98.31)	93 (88.57)
NTM	40 (3.89)	38 (4.40)	24 (3.57)	0 (0.00)	1 (0.42)	0 (0.00)
DST failed	12 (1.17)	37 (4.28)	6 (0.89)	7 (2.82)	3 (1.27)	12 (11.43)
**DST**	975	789	642	241	233	93
Susceptible	848 (86.97)	642 (81.37)	528 (82.24)	194 (80.50)	180 (77.25)	68 (73.12)
MDR-TB	32 (3.28)	34 (4.31)	32 (4.98)	17 (7.05)	22 (9.44)	11 (11.83)
XDR-TB	7 (0.72)	7 (0.89)	8 (1.25)	2 (0.83)	3 (1.29)	1 (1.08)
R-INH & S-RIF ^b^	46 (4.72)	38 (4.82)	28 (4.36)	10 (4.15)	14 (6.01)	10 (10.75)
R-RIF & S-INH ^c^	10 (1.03)	11 (1.39)	8 (1.25)	4 (1.66)	7 (3.00)	3 (3.23)
Other resistance	32 (3.28)	57 (7.22)	38 (5.92)	14 (5.81)	7 (3.00)	(0.00)

^a^M.tb: *Mycobacterium tuberculosis* with succeed DST.

^b^R-INH & S-RIF: resistant to isoniazid but susceptible to rifampicin.

^c^R-RIF & S-INH: resistant to rifampicin but susceptible to isoniazid.

From 2013 to 2015, a total of 176 MDR-TB and extensively drug-resistant tuberculosis (XDR-TB) cases were detected, with 120 cases in Prefecture E, and 56 cases in Prefecture W. The proportion of MDR-TB and XDR-TB cases in 2013, 2014, and 2015 was, respectively, 4.00%, 5.20%, and 6.23% in Prefecture E and 7.88%, 10.73%, and 12.91% in Prefecture W amongst cases with culture-positive *Mycobacterium tuberculosis* (*M.TB*) isolates and successful DST. This proportion was significantly higher in the western site than in the eastern site from 2013 to 2015 (2013: *p* = 0.012; 2014: *p* = 0.003; 2015: *p* = 0.022) (Table 2).

### Detected proportion of MDR/XDR-TB cases in the five high-risk groups

**Figure 1.**
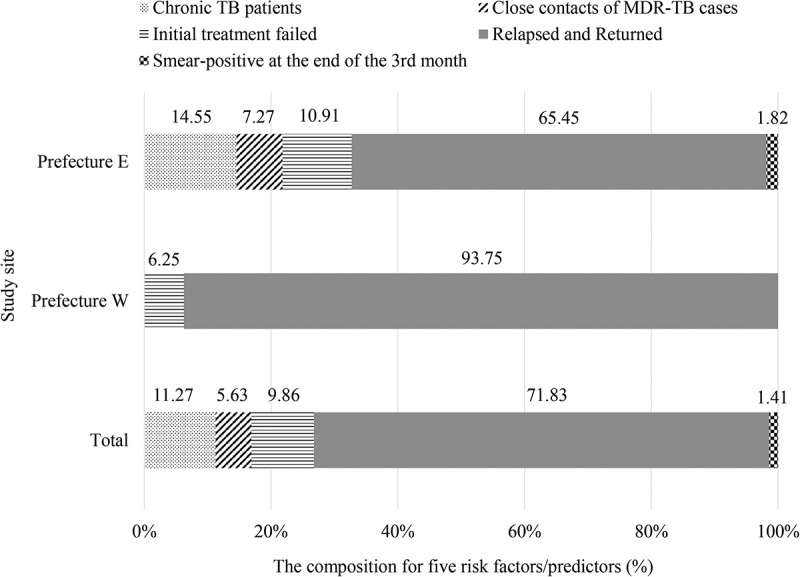
Prevalence of five factors/predictors amongst 71 MDR/XDR-TB patients who were from high-risk groups.

**Figure 2.**
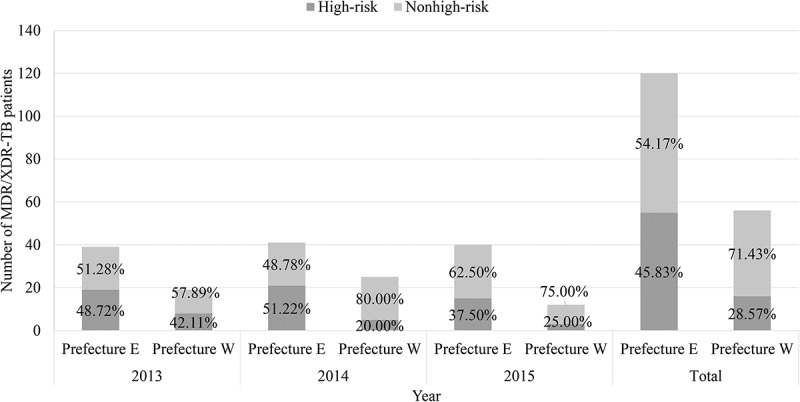
Proportion of detected MDR/XDR-TB patients based on the high-risk patient screening strategy from 2013 to 2015. The height of each stacked bar shows the absolute number of MDR/XDR-TB patients. The percentage in the dark grey rectangle represents the proportion of MDR/XDR-TB patients from the five high-risk groups, whereas the percentage in the light grey rectangle represents the proportion of patients who were not from the five high-risk groups.

Overall, 40.34% (71/176) of the MDR/XDR-TB patients were from the five high-risk groups. Of these 71 MDR/XDR-TB cases from high-risk groups, their composition in groups 1 to 5 was 11.27%, 5.63%, 9.86%, 71.83%, and 1.41%, respectively (Figure 1). The proportion of MDR/XDR-TB cases detected from the five high-risk groups was significantly higher in Prefecture E than in Prefecture W (Prefecture E: 45.83%, 55/120; Prefecture W: 28.57%, 16/56, *p* = 0.021). There were no significant differences amongst the years with regard to the proportion of MDR-TB patients detected from high-risk groups in both sites (Prefecture E: *p* = 0.421; Prefecture W: p from Fisher’s exact test: 0.322) (Figure 2).

### PAR% for the five risk factors/predictors

After adjusting for age and sex, the TB patients from the five high-risk groups were more likely to have MDR-TB than those from the low-risk groups, with an aOR ranging from 2.62 (95% CI: 0.87–7.90) to 11.06 (95% CI: 5.45–22.44), except in 2015 in Prefecture W. Although there was a high risk of MDR-TB in these five groups, the large number of low-risk patients contributed significantly to the number of MDR-TB cases, which led to a PAR% for all five high-risk groups of 43.4% (95% CI: 24.6–61.7%), 49.9% (95% CI: 31.3–67.0%), and 30.3% (95% CI: 12.9–50.1%) in Prefecture E for 2013, 2014, and 2015, respectively, whereas the corresponding values were 36.6% (95% CI: 10.4–64.5%), 13.3% (95% CI: −1.7–39.7%), and −82.5% (95% CI: −117.5 to −11.2%) in Prefecture W, respectively. The negative PAR% value reflects a cOR< 1 for this calculation and the lack of contribution from high-risk groups to MDR-TB detection. Based on these results, less than half of MDR-TB patients belonged to the five high-risk groups (Table 3). The PAR% for the five specific risk factors/predictors ranged from 0.4% (95% CI: −0.2 to 4.8%) to 21.0% (95% CI: 13.1–30.0%) in these two prefectures during the study period (Table 4).

**Table 3.** Odds ratios and population attributable risk percentages for high-risk groups in prefecture E and prefecture W from 2013 to 2015.

Year	Site	Group ^a^	MDR/XDR *n* (%)	Non-MDR/XDR *n* (%)	cOR (95% CI)	aOR (95% CI) ^b^	PAR% (95% CI) ^c^
2013	Prefecture E	High-risk	19 (13.29)	124 (86.71)	6.22 (3.23–11.99)	7.61 (3.84–15.08)	43.4 (24.6–61.7)
		Low-risk	20 (2.40)	812 (97.60)			
	Prefecture W	High-risk	8 (21.05)	30 (78.95)	4.66 (1.73–12.51)	5.00 (1.79–13.93)	36.6 (10.4–64.5)
		Low-risk	11 (5.42)	192 (94.58)			
2014	Prefecture E	High-risk	21 (20.79)	80 (79.21)	8.77 (4.56–16.88)	11.06 (5.45–22.44)	49.9 (31.3–67.0)
		Low-risk	20 (2.91)	668 (97.09)			
	Prefecture W	High-risk	5 (20.83)	19 (79.17)	2.49 (0.84–7.38)	2.62 (0.87–7.90)	13.3 (−1.7–39.7)
		Low-risk	20 (9.57)	189 (90.43)			
2015	Prefecture E	High-risk	15 (15.96)	79 (84.04)	3.97 (2.01–7.86)	4.94 (2.42–10.09)	30.3 (12.9–50.1)
		Low-risk	25 (4.56)	523 (95.44)			
	Prefecture W	High-risk	3 (5.66)	50 (94.34)	0.21 (0.05–0.82)	0.22 (0.05–0.95)	−82.5 (−117.5- −11.2) ^d^
		Low-risk	9 (22.50)	31 (77.50)			

^a^High-risk: patients who were from the five high-risk groups; low-risk: patients who were not from the five high-risk groups.

^b^Adjusted odds ratio and 95% confidence interval: age and sex were adjusted.

^c^PAR% was calculated using the crude odds ratio and the exposure proportion of all five risk factors amongst MDR-TB cases.

^d^PAR% was negative because the crude odds ratio for the five risk factors was less than 1.00, which denoted a higher MDR-TB prevalence amongst the low-risk group than the high-risk group.

**Table 4.** Odds ratios and population attributable risk percentages for five risk factors.

		MDR/XDR	Non-MDR/XDR			
		*n* = 176	*n* = 2797			
Five risk factors ^a^		*n* (%)	*n* (%)	cOR (95% CI)	aOR (95% CI) ^b^	PAR% (95% CI) ^c^
Chronic TB patients	Yes	8 (72.73)	3 (27.27)	44.35 (11.66–168.70)	49.81 (12.89–192.53)	13.8 (3.8–38.3)
	No	168 (5.67)	2794 (94.33)			
Close contacts of MDR-TB cases	Yes	4 (13.79)	25 (86.21)	2.58 (0.89–7.49)	2.93 (1.00–8.62)	1.5 (−0.1–6.0)
	No	172 (5.84)	2772 (94.16)			
Initial treatment failure	Yes	7 (23.33)	23 (76.67)	5.00 (2.11–11.81)	5.14 (2.15–12.29)	3.9 (1.1–9.8)
	No	169 (5.74)	2774 (94.26)			
Relapsed and returned	Yes	51 (13.56)	325 (86.44)	3.10 (2.20–4.38)	3.61 (2.53–5.16)	21.0 (13.1–30.0)
	No	125 (4.81)	2472 (95.19)			
Smear-positive at the end of the third month	Yes	1 (14.29)	6 (85.71)	2.66 (0.32–22.20)	2.81 (0.33–24.01)	0.4 (−0.2–4.8)
	No	175 (5.90)	2791 (94.10)			

^a^Chronic TB patients: patients who experienced multiple treatment courses but remained sputum positive, or patients for whom retreatment failed. Close contacts of MDR-TB cases: close contacts of MDR-TB cases who developed sputum smear-positive TB. Initial treatment failed: new patients who were sputum smear-positive at the end of fifth month of treatment. Relapsed and returned: patients had a definite TB history but were cured after standard treatment and developed smear-positive sputum again in the current episode, or patients who had ≥1 month’s anti-TB treatment with ≥2 months’ interruption and returned to treatment again. Smear-positive at the end of the third month: patients who remained smear-positive at the end of the third month of an initial treatment regimen.

^b^Adjusted odds ratio and 95% confidence interval: age and sex were adjusted.

^c^PAR% was calculated using the crude odds ratio and the exposure proportion of the risk factor amongst MDR-TB cases.

## Discussion

The present study was the first to evaluate the effect of an MDR-TB detection screening strategy based on five high-risk groups in China. According to our estimates, at least half of the MDR-TB patients would not have been detected if only the five high-risk groups were screened in these two prefectures of China. Only 26% of MDR/RR-TB cases were detected globally in 2016, and China had the lowest case detection rate [1]. The high prevalence and low case detection rate have become a critical challenge preventing China from meeting the goal of ending the global TB epidemic by 2035 [1].

The Chinese government has expended tremendous efforts to prevent and control TB and MDR-TB by launching a series of policies in past decades [10]. The proportion of MDR-TB cases in the present study was 5% in Prefecture E and 10% in Prefecture W, which was lower than the proportion of incident MDR-TB cases in China (12%) based on a previous meta-analysis [19]. Meanwhile, a substantial spatial difference in the MDR-TB burden between Prefecture E and Prefecture W was observed in the current study, which was consistent with recent systematic reviews [19,20]. The difference in the prevalence of culture-positive TB and MDR-TB amongst diagnosed TB patients between the eastern and western sites might be due to social economic status, educational level, population density and mobility, proportion of ethnic minorities, and distribution of relevant diseases based on geographic features. TB has been viewed as a disease of the poor. The risk of developing MDR-TB is the highest amongst poor and/or vulnerable members of the population [10]. Most MDR-TB cases were located in the southwest and northeast regions, which are less-developed areas of China [21]. In these less-developed regions, a high burden of AIDS, plateau terrain, multi-ethnic inhabitants, and unfavourable conditions have constrained the resources for MDR-TB detection and prevention [21]. A survey performed in three prefectures of China in 2013 found that the annual funding allocated to TB control was much less in the prefecture located in the western region than in two prefectures in the eastern and central regions [22].

China is responsible for nearly 9.7% of the estimated 600,000 MDR/RR-TB cases worldwide. It is crucial that China improves the detection of MDR-TB and closes the detection gap for eliminating the disease [23]. Previous studies have shown that inadequate treatment of MDR-TB is a severe problem in China, particularly in hospitals [5,24]. Thus, it has become a common belief that patients who have a previous history of anti-TB treatment are most likely to develop drug resistance [13,25–27]. This study confirmed that the risk of developing MDR-TB was 2 to 11 times higher amongst TB patients who were in the five high-risk groups than amongst patients who were not. Nevertheless, the contribution of a high-risk screening strategy to MDR-TB detection was not sufficient considering the high number of incident TB patients (895,000 per year in China) [1].

The estimated PAR% for the five high-risk factors/predictors was only approximately 50%, and the PAR% was even lower in Prefecture W than in Prefecture E. This finding indicates that less than one-half of smear-positive MDR-TB patients are associated with the five risk factors/predictors. Other countries with a high MDR-TB burden have adopted relevant strategies for better detection and control of drug resistance. For example, South Africa, which is also a BRICS country, has provided universal coverage of DST [28]. Patients are treated as MDR-TB patients and referred to MDR-TB institutions for further diagnosis and treatment if they are diagnosed as RR-TB based on the GeneXpert MTB/RIF test or other rapid diagnostic method [28]. The number of laboratory-confirmed MDR-TB cases was greater than the estimated number of cases in South Africa [1]. In addition, India has adopted the GeneXpert assay in place of the cumbersome culture-based DST as the initial diagnostic test for people with presumptive TB since 2011, as recommended by the WHO, irrespective of the presence of risk factors for MDR-TB [29]. As a result, more than 500 Xpert MTB/RIF test centres were established in India by 2016 [29]. In the Russian Federation, more attention has been paid to creating new diagnostic tools, such as the Line Probe Assay, to reduce the time needed for an MDR diagnosis, to initiate treatment earlier and to promote better treatment outcomes for patients with MDR-TB [30]. China has the third largest TB burden globally, and only 10.5% to 15% of active cases are retreated [1,31]. The proportion the five high-risk groups was much smaller than the proportion of the low-risk groups amongst all TB cases, particularly amongst incident TB cases. Since 7.1% of new cases were estimated to have MDR/RR-TB in China, a substantial proportion of MDR-TB patients would not be detected unless some low-risk patients were also eligible for screening [1]. Therefore, it is essential to enhance access to rapid diagnostic tests and universal DST and to provide MDR-TB screening to both high-risk and low-risk patients for a more accurate diagnosis and treatment.

The influential Global Fund project for MDR-TB control ended its support for operations in China on 30 June 2014 [8,12]. The absolute number of smear-positive TB patients who received DST decreased substantially in 2015 compared with 2013 and 2014. Although the Global Fund has brought many invaluable gains to China’s fundamental approach to MDR-TB control, this programme could not provide a long-term solution to MDR/RR-TB control if China did not transform its role from an aid recipient into a full and active ‘End TB’ partner [10]. Resources in eastern China (Jiangsu Province) were subsequently mobilized to raise funding for TB/MDR-TB management, and appropriate incentives were given to both health facilities and health care providers after the end of the Global Fund. However, there was much less sustained government financing in western China (Sichuan Province), which might explain the decrease in DST after 2014. Thus, there was a lower prevalence of MDR-TB amongst high-risk patients than low-risk patients, leading to a PAR% < 0 in Prefecture W in 2015, which indicated that the possibility of detecting MDR-TB cases would be poor if DST was applied to only five high-risk groups. Economic success in China has generated substantial resources to address the MDR-TB crisis. The central and local governments of China must pick up where the Global Fund left off by increasing domestic funding to confront TB and MDR-TB.

Nevertheless, this study has limitations. First, this surveillance-based longitudinal data analysis included only notified sputum smear-positive TB cases. MDR/XDR-TB cases amongst smear-negative TB patients and TB patients who were not notified could not be detected. Therefore, the prevalence of MDR-TB might be underestimated. Second, the measurement of PAR is usually used in prospective cohort studies to estimate the effects of risk factors on the incidence of diseases. In this study, we calculated the PAR to estimate the contribution of high-risk screening to the detection of MDR-TB cases using longitudinal surveillance data considering that MDR/RR-TB is a rare disease in the population and that the surveillance is conducted temporally. Thus, the present study could estimate only the possibilities of detecting MDR-TB cases through screening high-risk groups in similar settings. Finally, the generalizability of these findings to other Chinese populations may be limited because the prevalence of MDR-TB amongst high- and low-risk groups varies based on the time, place, and population.

## Conclusions

The five high-risk groups had higher risks of developing MDR-TB. However, less than half of the detected smear-positive MDR-TB patients were associated with the five risk factors. Consequently, this high-risk screening strategy would miss a substantial portion of MDR-TB patients who do not belong to the five high-risk groups, such as newly diagnosed patients without previous anti-TB treatment. These undetected cases would remain the source of transmission for a long time and prolong the epidemic of MDR-TB. For better detection and control of MDR-TB in China, an increase in domestic funding is urgently required to expand the coverage of DST with conventional methods and rapid tests.

## Paper context

Although high-risk groups had a higher risk of developing MDR-TB, less than half of the detected MDR-TB patients were associated with five risk factors in China. A high-risk screening strategy would miss a substantial part of the MDR-TB cases who did not belong to five high-risk groups, such as newly diagnosed patients without previous anti-TB treatment. Increasing domestic funding and expanding the coverage of DST screening is urgently required to confront MDR-TB in China.
